# Can the low-carbon city pilot policy promote firms’ low-carbon innovation: Evidence from China

**DOI:** 10.1371/journal.pone.0277879

**Published:** 2023-01-11

**Authors:** Ge Yang

**Affiliations:** Economics and Management School, Wuhan University, Wuhan, China; Instituto Tecnologico Autonomo de Mexico, MEXICO

## Abstract

The low-carbon city pilot policy is an environmental regulation aimed at reducing carbon emissions at the municipal level. Previous research mostly focused on evaluating its environmental performance and discovered it could enhance pilot cities’ low-carbon innovation. However, the effects of the low-carbon city pilot policy on firm-level low-carbon innovation and their economic impact have yet to be investigated. This research uses a sample of Chinese A-share listed firms and the difference-in-difference method to examine the effect of the low-carbon city pilot policy on firms’ low-carbon innovation. The baseline regression showed that the low-carbon city pilot policy could greatly encourage low-carbon innovation among firms in pilot cities. The mechanism analysis demonstrated that this improvement effect is attained by easing these firms’ financing constraints. According to the heterogeneity analysis, we discovered that state-owned firms and firms situated in pilot zones with municipal secretaries who have larger promotion incentives are more susceptible to this policy. Additionally, the research on this policy’s economic impact revealed that, following its adoption, the market value and comparative advantages of the firms in the pilot areas also increased. The findings of this study have implications for the enhancement and national expansion of low-carbon policies adopted at the city level.

## Introduction

With the dramatic increase in global greenhouse gas emissions, climate change has become one of the world’s significant challenges today. The Paris Agreement clarifies the world’s commitment to keeping the rise in average global temperature to 2 degrees Celsius, with intentions to reduce it to 1.5 degrees Celsius by the end of this century. While China’s overall energy consumption expanded from 602 million tons of standard coal in 1980 to 4.86 billion tons in 2019, the massive energy consumption has resulted in a considerable quantity of carbon emissions, making China the world’s greatest energy consumer and carbon emitter. The Chinese government promised at the Copenhagen Climate Conference in 2009 to cut carbon emission intensity by 40% to 45% by 2020 compared to 2005. It also pledged to peak its carbon emissions by 2030 and attain carbon neutrality by 2060 during the UN General Assembly in September 2020. The targets for cutting carbon emissions are both a barrier to China’s future economic growth and a crucial window of opportunity for the country to move toward a low-carbon economy.

To meet the carbon emission reduction objective, the Chinese government has implemented several environmental regulations to minimize greenhouse gas emissions. The low-carbon city pilot policy is one of them. This strategy was implemented by China’s National Development and Reform Commission in three batches of provinces and cities in 2010, 2012, and 2017 to encourage the growth of low-carbon industries, the construction of low-carbon cities, and the advocacy of low-carbon lifestyles.

Since its launch, China’s low-carbon city pilot policy has drawn significant attention at home and internationally. The existing studies mainly evaluated the implementation effects of this policy and obtained some valuable findings. Studies at the city-level discovered that the low-carbon city pilot policy could significantly contribute to the upgrading of industrial structure [1], the conservation of energy [2–4], the reduction of emissions [5–8], and the improvement of green or low-carbon innovation [9–11] of pilot areas. In contrast, studies at the firm level found that this policy can reduce not only firms’ coal consumption and coal intensity [12] but also enhance firms’ green innovation [13, 14] and total factor productivity [15].

As an environmental policy aimed at reducing carbon emissions, whether the low-carbon city pilot policy can achieve the environmental objectives without affecting the competitive advantage or market value of firms is a question worth exploring. Previous studies suggest that the low-carbon pilot city policy can promote low-carbon innovation in China’s prefecture-level cities by influencing the innovation environment and environmental information disclosure [11]. However, the impacts of this policy on firms’ low-carbon innovation and its economic effect have yet to be studied.

Considering that firms are the main force in carrying out innovation activities and that low-carbon innovation has a larger initial investment and higher risk compared with traditional technology innovation, this paper aims to evaluate the impact of low-carbon pilot city policy on firms’ low-carbon innovation and reveal the intermediate mechanism of this effect. Besides, it will also investigate whether the implementation of the low-carbon city pilot policy can enhance firms’ economic performance at the same time if it does improve their low-carbon innovation.

## Policy background and research hypothesis

### Policy background

Low-carbon city pilot policy is an environmental policy implemented at the city level to establish a green and low-carbon economic development pattern in China. In 2010, the National Development and Reform Commission of China issued the Notice on the Piloting of Low-Carbon Provinces and Low-Carbon Cities. It launched the first batch of low-carbon pilots in 5 provinces and 8 cities. During the 12th Five-Year Plan period, China’s energy consumption per unit of GDP fell by 18.2%, and the proportion of non-fossil energy consumption rose by 2.6%. Additionally, from 2010 to 2011, CO2 emissions per unit of GDP in the pilot cities decreased by about 88.9% compared to other cities in the same province. The NDRC launched the second batch of pilots in 2013 and expanded the scope of low-carbon city pilots to 29 areas. In 2016, China’s energy consumption per unit of GDP fell by 5%, and CO2 emissions fell by approximately 7%, exceeding the annual targets set previously. Considering the positive results of the first two batches of pilots, the NDRC implemented the third batch of low-carbon pilot policies in 2017, covering 45 cities.

The low-carbon city pilot policy mainly has five objectives. The first one is to adjust the industrial structure of the pilot areas through technological transformation, thus achieving the upgrading of the industrial system in a low-carbon way and promoting the development of a circular economy. The second one is to adjust the pilot areas’ energy structure and encourage firms to increase the use of green and clean energy, thus reducing the use of primary energy and the emission of greenhouse gas. The third objective is to develop a low-carbon public urban transportation system, especially an electric bus system, thus minimizing the number of private cars and reducing carbon emissions. The fourth objective is to promote energy conservation in the building sector to realize the harmonious coexistence between humans and nature. The fifth objective is to instruct the pilot areas to establish their greenhouse gas data systems, compile their regional greenhouse gas emissions records, and build their carbon emissions trading markets.

Instead of setting specific targets, such as the peak time of carbon emissions and emission standards of different industries for every pilot city, the central government delegated this power to the local governments of the pilot cities so that the latter could carry out the work according to their situation. Compared with other environmental regulations with strict and unified planning, the low-carbon city pilot policy is an exploratory attempt. It remains to be tested whether weakly binding environmental regulation can obtain the desired effects.

### Research hypothesis

#### Low-carbon city pilot policy and low-carbon innovation

From the perspective of firms, achieving low-carbon production requires either reducing carbon emissions or increasing energy consumption efficiency, which may have two different effects on firms’ behavior. On the one hand, efforts to reduce carbon emissions will raise production costs and reduce the funds available for R&D, thus impeding firms’ potential to increase low-carbon innovation. On the other hand, since low-carbon innovation can help reduce firms’ energy intensity and energy consumption, and those with higher low-carbon innovation can produce qualified products with minimal environmental cost, firms affected by related environmental regulations may have a higher willingness to carry out low-carbon innovation. Therefore, the main hypothesis of this paper is proposed as follows:

H1a: China’s low-carbon city pilot policy can promote firms’ low-carbon innovation.

H1b: China’s low-carbon city pilot policies will inhibit firms’ low-carbon innovation.

#### The mediation effect of financing constraint

The R&D activities in firms are generally risky and involve a long time, making them susceptible to financing constraints. Financing constraint refers to the situation when a firm wants to increase investment but has insufficient internal resources and limited access to external capital market funding. Compared with traditional technological innovation, the initial investment in low-carbon innovation is larger, and its risk level is higher. Firms may be more likely to face financing constraints when undertaking low-carbon innovation.

Although the low-carbon city pilot policy is weakly binding, the central government seriously considered the pilot cities. Public officials in pilot cities would regard it as an honor and a chance, especially when the political promotion competition among officials is getting increasingly fierce. As a result, governments in pilot areas may have a higher incentive to encourage firms to engage in low-carbon production. We can see that these local governments have introduced various green financing policies, including special funds, subsidies, preferential interest rate loans and tax incentives for low-carbon projects. These green financing policies can alleviate firms’ financing constraints and promote them to increase R&D investment and improve their low-carbon innovation. The second hypothesis of this paper is proposed as follows:

*H2*: *China’s low-carbon city pilot policy can promote firms’ low-carbon innovation by alleviating their financing constraints*.

## Data and method

### Sample selection and core variables

#### Sample selection

In order to empirically tests the impact of China’s low-carbon city pilot policy on low-carbon innovation of firms, the sample used in this paper is all Chinese A-share listed firms in the industrial sector, and the sample interval is set from 2008 to 2016.

In the data cleaning process, samples with missing data and suffering serious losses (Marked as ST or *ST) are eliminated. Besides, all control variables are winsorized at 1% to avoid the influence of extreme values on the estimation results.

Considering that the third batch of pilot cities was just launched in 2017, and the impact of the low-carbon city pilot policy on firms in the first batch of pilot cities cannot be effectively observed before the launch of the second batch of pilot cities, this paper combines the first two batches of pilot cities in order to identify the net impact of low-carbon city pilot policy on firms’ low-carbon innovation. Referring to Xu and Cui (2020), 2012 is set as the starting point of this policy [16], and firms in the first two batches of pilot regions are divided into the treatment group, while firms in non-pilot regions are divided into the control group.

#### Core variables

*(1) Low-carbon innovation*. Due to the stability and objectivity of patent grant standards and the availability of relevant data, the number of patents is a very reliable indicator to reflect firms’ innovation level. This paper uses the number of firms’ low-carbon patents to measure their low-carbon innovation. Low-carbon patents are green technologies or applications that will mitigate or adapt to climate change. Based on the international patent classification codes of the Green Patent List released by the World Intellectual Property Organization, patents related to climate mitigation are considered low-carbon patents. They mainly include alternative energy production, energy conservation, carbon capture and storage, nuclear power generation, waste utilization, administrative regulation and design related to climate change mitigation. The low-carbon patent data of listed enterprises are obtained from the China Intellectual Property Office, and it is expressed as *lnl*_*ci*_ in this paper after adding one and taking the logarithm.

*(2) Financing constraint*. There are many ways to measure financing constraints, but most rely on endogenous financial indicators, which may bias the research conclusions. To avoid this deficiency, Hadlock and Pierce (2010) designed the SA index, which is constructed from two completely exogenous variables. The larger the absolute value of SA index is, the more serious the financing constraint firms face [17]. The SA index is calculated as follows:

$$SA=-0.737\times lncap+0.043\times {lncap}^{2}-0.04\times age$$ (1)

where *lncap* denotes the logarithm of firm size and *age* denotes firm age. Following Wu and Huang (2017) [18], this paper uses the logarithm of the absolute value of the SA index to measure a firm’s financing constraint.

#### Control variables

*Firm size*. When taking innovation as output and capital and labor as input, it is generally believed that the larger the firm size, the more innovation it will have according to the basic production function of the firm. Therefore, the total assets of the sample firm and their number of employees are selected as control variables, and they are expressed as *lnasset* and *lnlabor* after taking the logarithm.

*Firm maturity*. Barasa et al. (2017) found that firm age will affect its innovation output because mature firms can effectively absorb cutting-edge knowledge and technology [19]. So firm age is selected as a control variable to measure a firm’s maturity in this paper and is expressed as *lnage* after taking the logarithm.

*Firm profitability*. Zhang et al. (2020) suggest that a firm’s profitability can affect its R&D investment. The more profitable a firm is, the more R&D investment it may make and the more innovation it may have [20]. Therefore, the return on assets, the ratio of total operating income to total assets of the firm, is chosen as a control variable to measure firm profitability. This variable is expressed as *lnroa* after taking the logarithm.

*Debt status*. The debt status of a firm reflects the market’s evaluation of its credibility. Moderate debt enables a firm to carry out innovative activities such as equipment transformation and process upgrading with sufficient funds. In this paper, the logarithm of the ratio of the loan amount to total assets in the current year is used to measure a firm’s debt status and is denoted as *lndar*.

The descriptive statistics of the main variables are shown in Table 1 below.

**Table 1:** Descriptive statistics of main variables.

*VARIABLES*	*Obs*	*Mean*	*Std*. *Dev*.	*Min*	*Max*
*lnlci*	12,208	0.3955	0.7241	0	6.7696
*lnsa*	12,208	1.3141	0.3898	-4.2191	2.5905
*lnasset*	12,208	21.8944	1.2749	16.1167	28.5087
*lnlabor*	12,208	7.71792	1.2594	2.0794	13.2227
*lnage*	12,208	2.5498	0.4632	0	3.5835
*lndar*	12,208	-1.0482	0.6889	-4.9505	4.5742
*lnroa*	12,208	-0.61261	0.6359	-4.4085	3.1326

### Model setting

#### DID model

This paper uses the difference-in-difference model to analyze the impact of low-carbon city pilot policy on firms’ low-carbon innovation. Although this method was first proposed in the field of natural sciences, it has become one of the most commonly used methods for policy evaluation since Ashenfelter (1978) introduced it into economics studies [21]. The basic idea behind this method is to treat a new policy issued by the government as a natural experiment exogenous to the economic system. It identifies the average treatment effect of the policy by the difference between those who are affected and those who are not before and after the introduction of the policy. Compared with traditional policy evaluation methods, the difference-in-difference approach has the advantage of alleviating the problems caused by endogeneity and omitted variables. The difference-in-difference model used in this paper is constructed as follows:

$${lnlci}_{it}=\alpha_{0}+\alpha_{1}{pilot}_{i}\times {post}_{t}+\alpha_{2}X_{it}+\mu_{i}+\gamma_{t}+\varepsilon_{it}$$ (2)

where the subscript *i* represents the individual (firm), and *t* represents the time (year). The explanatory variable *lnlci*_*it*_ is the low-carbon innovation of firm *i* in year *t*. *pilot*_*i*_ is a dummy variable which takes 1 if firm *i* is in the pilot cities and takes 0 if firm *i* is in other areas. *post*_*t*_ is also a dummy variable, and it takes 1 if *t*≥2012 and takes 0 if *t*<2012. *X*_*it*_ represents all control variables. *μ*_*i*_ and *γ*_*t*_ denote firm fixed and time fixed effects, respectively, and *ε*_*it*_ is a stochastic disturbance term. In addition, *pilot*_*i*_*×post*_*t*_ is the core variable of this paper, whose coefficient reflects the impact of low-carbon city pilot policy on firms’ low-carbon innovation.

#### Mediation models

In order to explore how the influence of low-carbon city pilot policy on firms’ low-carbon innovation is realized, this paper uses mediation models to analyze the mediator role of firms’ financing constraints. The mediation models are set as below:

$${lnsa}_{it}=\beta_{0}+\beta_{1}{pilot}_{i}\times {post}_{t}+\beta_{2}X_{it}+\mu_{i}+\gamma_{t}+\varepsilon_{it}$$ (3)

$${lnlci}_{it}=\delta_{0}+\delta_{1}{pilot}_{i}\times {post}_{t}+\delta_{2}{lnsa}_{it}+\delta_{3}X_{it}+\mu_{i}+\gamma_{t}+\varepsilon_{it}$$ (4)

Eqs (3) and (4) are quite similar to Eq (2). However, the explained variable in Eq (3) is *lnsa*_*it*_, which is the financing constraint faced by firm *i* in year *t*, and Eq (4) has an additional explanatory variable *lnsa*_*it*_ on the right side compared with Eq (1).

If *α*_*1*_ in Eq (1) is significant, this paper will adopt the stepwise regression method to test the mediation effect of financing constraints. The general steps are as follows: if *β*_*1*_ in Eq (3) is insignificant, it indicates that the causal relationship between the low-carbon city pilot policy and the firm’s financing constraint is weak or does not exist, then there is no need to regress Eq (4). If *β*_*1*_ is significant and so do *δ*_*1*_ and *δ*_*2*_ in Eq (4), and at the same time *δ*_*1*_ is closer to 0 than *α*_*1*_, it means that the low-carbon city pilot policy can affect firms’ low-carbon innovation through its influence on firms’ financing constraints.

## Empirical analyses and results

### Benchmark regression results

This paper empirically tests the impact of the implementation of a low-carbon city pilot policy on low-carbon innovation of Chinese firms based on Eq (1), and the results are shown in columns (1) to (6) in Table 2. Column (1) does not include any control variables, and these variables are gradually added in columns (2) to (6). It could be found that difference-in-difference coefficients in all columns are significantly positive at the 1% level, indicating that after the implementation of the low-carbon city pilot policy, the low-carbon innovation of firms has been significantly improved. Specifically, the coefficient of *pilot×post* in column (6) is 0.585, meaning that the low-carbon city pilot policy increases the low-carbon innovation of firms in pilot areas (treated group) by 0.585 compared to firms in other areas (control group). Therefore, hypothesis H1a is proved.

**Table 2:** Baseline regression results.

	**(1)**	**(2)**	**(3)**	**(4)**	**(5)**	**(6)**
	*lnlci*	*lnlci*	*lnlci*	*lnlci*	*lnlci*	*lnlci*
*pilot×post*	0.589***	0.589***	0.586***	0.586***	0.586***	0.585***
	(0.174)	(0.174)	(0.175)	(0.175)	(0.175)	(0.175)
*lnasset*		0.0339***	0.0104	0.0101	0.0101	0.00264
		(0.00934)	(0.0112)	(0.0112)	(0.0112)	(0.0113)
*lnlabor*			0.0332***	0.0314***	0.0313***	0.0386***
			(0.00936)	(0.00932)	(0.00940)	(0.00959)
*lnage*				0.101***	0.100***	0.102***
				(0.0375)	(0.0379)	(0.0379)
*lndar*					0.00088	0.00173
					(0.0101)	(0.0101)
*lnroa*						-0.0329***
						(0.0117)
time fixed effects	YES	YES	YES	YES	YES	YES
firm fixed effects	YES	YES	YES	YES	YES	YES
observations	12,208	12,208	12,208	12,208	12,208	12,208
R-squared	0.753	0.753	0.754	0.754	0.754	0.754

Note: Robust standard errors are in parentheses. ***, **, and * indicate the significance levels of 1%, 5% and 10% respectively.

In control variables, the coefficients of employee number, firm age and firm profitability are all significantly positive at 1%, reflecting that firms with more employees, longer development time and higher profitability will have more green-carbon innovation. Nevertheless, for other control variables, no statistically significant correlation is observed between them and the firm’s green-carbon innovation.

### Robustness tests

#### Parallel trend test and placebo test

A prerequisite for using the difference-in-difference approach is that the parallel trend hypothesis is satisfied. This means that before the introduction of the low-carbon city pilot policy, firms in the pilot areas (treatment group) and other areas (control group) have the same trend in developing low-carbon innovations. This paper constructs the following model to test the parallel trend hypothesis:

$${lnlci}_{it}=\theta_{0}+\sum_{t=2009,t\neq 2011}^{2016}\theta_{t}{pilot}_{i}\times d_{t}+\theta_{1}X_{it}+\mu_{i}+\gamma_{t}+\varepsilon_{it}$$ (5)

where *d*_*t*_ is the year dummy variable, and if the year is 2009, *d*_*2009*_ = 1 and the rest are 0. To avoid complete collinearity, the year 2011 is excluded. Theoretically, the difference-in-difference model can satisfy the parallel trend hypothesis if *θ*_*2009*_ and *θ*_*2010*_ are not significant while *θ*_*2012*_ to *θ*_*2016*_ are all significant. Columns (1) and (2) of Table 3 are the regression results of the parallel trend test, with no control variables added in column (1) and all control variables added in column (2). We can see that the coefficients of *pilot×d*_*2009*_ and *pilot×d*_*2010*_ are not significant, but the coefficients of the year after that are all significantly positive, which is consistent with the parallel trend hypothesis.

**Table 3:** Parallel trend test and placebo test.

	(1)	(2)	(3)	(4)	(5)
	parallel trend	parallel trend	*pilot×d2009*	*pilot×d2010*	*pilot×d2011*
	*lnlci*	*lnlci*	*lnlci*	*lnlci*	*lnlci*
*pilot×post*			-0.0413	-0.0229	-0.0177
			(0.0277)	(0.0246)	(0.0281)
*pilot×d2009*	-0.0451	-0.0452			
	(0.0454)	(0.0449)			
*pilot×d2010*	-0.0613	-0.0612			
	(0.0404)	(0.0402)			
*pilot×d2012*	0.542*	0.542*			
	(0.301)	(0.301)			
*pilot×d2013*	0.538***	0.537***			
	(0.176)	(0.174)			
*pilot×d2014*	0.556***	0.553***			
	(0.184)	(0.181)			
*pilot×d2015*	0.561***	0.556***			
	(0.191)	(0.188)			
*pilot×d2016*	0.549***	0.542***			
	(0.105)	(0.103)			
control variables	NO	YES	YES	YES	YES
time fixed effects	YES	YES	YES	YES	YES
firm fixed effects	YES	YES	YES	YES	YES
observations	12,208	12,208	4,117	4,117	4,117
R-squared	0.753	0.754	0.781	0.781	0.781

Note: Robust standard errors are in parentheses. ***, **, and * indicate the significance levels of 1%, 5% and 10% respectively.

Moreover, this paper also conducts a placebo test, assuming that the low-carbon city pilot policy was implemented in 2009, 2010, and 2011 respectively, and performs difference-in-difference regressions after removing the samples of 2012 and beyond. The results are presented in Columns (3) to (5) in Table 3. We could find that all the core coefficients are insignificant, indicating that the results obtained in this paper are robust.

#### Eliminating the impact of important events

In the sample period of this paper, some concurrent events, such as the Global Financial Crisis, Beijing Olympic Games and environmental regulation policies in some regions, may also affect the low-carbon innovation of firms. In order to reduce the bias caused by ignoring these events, this paper deletes some samples and re-run the regression.

The Global Financial Crisis of 2008–2009 had a significant impact on China’s economic growth, which may affect the economic performance of firms and reduce their low-carbon innovation. In order to exclude their influence on the benchmark regression results, samples in 2008 and 2009 were removed, and the results of the new regression are shown in columns (1) and (2) of Table 4. We can see no significant change in the estimated results compared with that in Table 2.

**Table 4:** Eliminating the impact of important events.

	(1)	(2)	(3)	(4)	(5)	(6)
	Global Financial Crisis		Beijing Olympic Games		Other environmental policies	
	*lnlci*	*lnlci*	*lnlci*	*lnlci*	*lnlci*	*lnlci*
*pilot×post*	0.600***	0.597***	0.543***	0.544***	0.573***	0.581***
	(0.209)	(0.210)	(0.193)	(0.193)	(0.180)	(0.182)
control variables	NO	YES	NO	YES	YES	YES
time fixed effects	YES	YES	YES	YES	YES	YES
firm fixed effects	YES	YES	YES	YES	YES	YES
province×year fixed effects	NO	NO	NO	NO	YES	NO
city×year fixed effects	NO	NO	NO	NO	NO	YES
observations	10,411	10,411	10,388	10,388	12,208	12,208
R-squared	0.783	0.784	0.716	0.717	0.754	0.754

Note: Robust standard errors are in parentheses. ***, **, and * indicate the significance levels of 1%, 5% and 10% respectively.

During the 2008 Beijing Olympic Games, Beijing and its neighboring cities jointly carried out a series of environmental protection projects to achieve the idea of green Olympics. These projects may influence the low-carbon innovation of firms in these regions. So, to obtain the net impact of the low-carbon city pilot policy on firms’ low-carbon innovation, firms in Beijing, Tianjin, Hebei, Shanxi, Inner Mongolia and Liaoning are removed from the sample. The regression results are shown in columns (3) and (4) of Table 4, and it can be seen that they remain basically unchanged.

In 2013, a carbon trading pilot policy was implemented in seven regions, including Beijing, Tianjin, Shanghai, Hubei, Chongqing, Guangdong and Shenzhen. If the influence of this policy is not excluded, the conclusion of the benchmark regression in this paper may be biased. Therefore, referring to Chen (2020) [22], this paper added the cross-product term of province fixed effects and year fixed effects into model (1). The regression results are shown in column (5) of Table 4, and the coefficient of the core variable remains significantly positive. Besides, considering that National Ambient Air Quality Standards established in 2012 may also affect firms’ low-carbon innovation, a cross-product term of city fixed effects and year fixed effects is added to Eq (1). The regression results are shown in column (6) of Table 4, and we can see that the core variable is still significantly positive.

The above tests and results prove the robustness of the results of this paper.

#### Heterogeneity analysis

With the aim of verifying the effectiveness of the low-carbon city pilot policy, this paper analyses the heterogeneity of the impact of this policy from the perspectives of the city and firm.

In terms of the differences between cities, as the central government gradually increases the weight of environmental governance in the assessment of local officials, the policy effect of the low-carbon city pilot policy is likely to be affected by the promotion incentives of local officials. Previous studies have found that the age of government officials is an important factor affecting their promotion opportunities. The older an official is, the smaller his promotion incentive will be [23]. Therefore, in this paper, the age of the city’s municipal secretary is used to represent the degree of official promotion incentive. According to the median age of municipal secretaries in the sample in 2011, the year before the implementation of the low-carbon city pilot policy, the samples are divided into a strong promotion incentive group and a weak promotion incentive group. The regression results are listed in column (1) and column (2) of Table 5, with column (1) showing the regression results of the weak promotion incentive group and column (2) showing the regression results of the strong promotion incentive group. It can be found that the difference-in-difference coefficient in column (1) is not significant, while that in column (2) is significantly positive at the 1% level. These results show that the stronger the promotion incentive of local officials, the more attention they will pay to environmental governance in their jurisdiction, which will promote the implementation of the low-carbon city pilot policy, thus improving the low-carbon innovation of local firms to a greater extent.

**Table 5:** Heterogeneity analysis.

	(1)	(2)	(3)	(4)
	Weak promotion incentives	Strong promotion incentives	Non-state-owned firms	State-owned firms
	*lnlci*	*lnlci*	*lnlci*	*lnlci*
*pilot×post*	0.489	0.599***	0.309	0.604***
	(0.383)	(0.208)	(0.223)	(0.213)
control variables	YES	YES	YES	YES
year fixed effects	YES	YES	YES	YES
firm fixed effects	YES	YES	YES	YES
observations	4,406	7,802	7,320	4,888
R-squared	0.676	0.776	0.735	0.776

Note: Robust standard errors are in parentheses. ***, **, and * indicate the significance levels of 1%, 5% and 10% respectively.

In terms of the differences between firms, considering that firms with different ownership types are affected by government intervention to different degrees, this paper divides the samples into a state-owned firms group and a non-state-owned firms group. Detailed regression results are listed in columns (3) and (4) of Table 5, where column (3) shows the regression results for the non-state-owned firms’ group and column (4) shows the regression results for the state-owned firms’ group. It can be found that the difference-in-difference coefficient in column (3) is insignificant, while that in column (4) is significantly positive at the 1% level, showing that the low-carbon city pilot policy has a greater impact on state-owned firms. The possible reasons behind this result are that state-owned firms have taken on more political tasks and are more responsive to environmental policies. In contrast, non-state-owned firms are more sensitive to changes in production costs. To maximize short-term profits, they can choose other ways to reduce costs and increase low-carbon innovation, such as relocating to areas with relatively relaxed environmental regulations. Therefore, compared with non-state-owned firms, the low-carbon city pilot policy has a greater impact on the low-carbon innovation of state-owned firms.

### Mechanism analysis

Based on Eqs (3) to (5), a mediation effect analysis is conducted to explore whether a low-carbon city pilot policy can promote low-carbon innovation by alleviating firms’ financing constraints. The regression results are shown in columns (1) and (2) of Table 6. In column (1), the coefficient of *pilot×post* is significantly negative, indicating that the financing constraint of firms in pilot areas could be alleviated. Meanwhile, the coefficient of *pilot×post* is significantly positive, and that of *lnsa* is significantly negative in column (2), reflecting that financing constraint plays a significant role in mediating the positive relationship between low-carbon city pilot policy and firm’ low-carbon innovation. Therefore, Hypothesis 2 is proved.

**Table 6:** Mechanism analysis.

	(1)	(2)
	*lnsa*	*lnlci*
*pilot×post*	-0.32***	0.586***
	(0.0502)	(0.175)
*lnsa*		-0.216***
		(0.044)
control variables	YES	YES
year fixed effects	YES	YES
firm fixed effects	YES	YES
firm-specific time trend	YES	YES
observations	12,208	12,208
R-squared	0.860	0.754

Note: Robust standard errors are in parentheses. ***, **, and * indicate the significance levels of 1%, 5% and 10% respectively.

### Economic effects of low-carbon city pilot policy

The above analysis concludes that the low-carbon city pilot policy can effectively promote the low-carbon innovation of firms. However, will the low-carbon innovation of firms under this policy reduce their economic performance or bring them substantial economic returns? This paper further investigates whether implementing the low-carbon city pilot policy will improve firms’ competitive advantage and market value through the channel of low-carbon innovation, and the following model was constructed:

$${Performance}_{it}=\vartheta_{0}+\vartheta_{1}{lnlci}_{it}+\vartheta_{2}{pilot}_{i}\times {post}_{t}\times {lnlci}_{it}+\vartheta_{3}{pilot}_{i}\times {post}_{t}+\vartheta_{4}{lnlci}_{it}\times {post}_{t}+\vartheta_{5}{lnlci}_{it}\times {pilot}_{i}+\vartheta_{6}X_{it}+\mu_{i}+\gamma_{t}+\varepsilon_{it}$$ (6)

In Eq (6), *performance* is the explanatory variable representing market performance, which includes product-market competitive advantage *opm* and firm market value *tobinq*. Product-market competitive advantage *opm* is measured by the ratio of gross profit to operating revenue, and the market value of a firm *tobinq* is measured by the ratio of the market value of a firm to its replacement cost of capital.

The detailed regression results are shown in Table 7. The explanatory variables in columns (1) and (2) are *opm* and the explanatory variables in columns (3) and (4) are *tobinq*. It could be found that the coefficients of the triple interaction terms are significantly positive in all columns, which indicates that the low-carbon city pilot policy can improve firms’ low-carbon innovation, thus improving their market value and the competitive advantage of their products. The reason may be that low-carbon innovation can act as a signal to win firms the approval of investors and customers. This result also shows that the low-carbon city pilot policy can achieve the purpose of protecting the environment and generating considerable economic returns at the same time.

**Table 7:** Economic effects of low-carbon city pilot policy.

	(1)	(2)	(3)	(4)
	*opm*	*opm*	*tobinq*	*tobinq*
*pilot×post×lnlci*	0.157**	0.108*	0.071**	0.0148*
	(0.0677)	(0.061)	(0.0304)	(0.00795)
control variables	NO	YES	NO	YES
year fixed effects	YES	YES	YES	YES
firm fixed effects	YES	YES	YES	YES
observations	12,208	12,208	12,208	12,208
R-squared	0.551	0.635	0.847	0.854

Note: Robust standard errors are in parentheses. ***, **, and * indicate the significance levels of 1%, 5% and 10% respectively.

## Conclusions and implications

This paper found that after implementing the low-carbon city pilot policy, the low-carbon innovation of firms in pilot areas has been significantly improved compared with that in non-pilot areas. This result extends earlier studies which only explore the impact of the low-carbon city pilot policy on the low-carbon innovation at the city level. From the firm perspective, we found that the impact of this policy on firms’ low-carbon innovation is achieved by alleviating their financing constraints, and state-owned firms and firms located in pilot areas whose municipal secretary has stronger promotion incentives are more sensitive to this policy. Besides, previous studies on the low-carbon city pilot policy mainly focused on its impact on environmental performance without considering its economic consequences. Through the analysis of economic effects, we found that the market value and comparative advantages of firms in pilot areas have also improved after this policy’s implementation. Our results show that the low-carbon pilot city policy is a win-win policy that can achieve both environmental protection and economic benefits.

The policy implication of this study is that the low-carbon city pilot policy is worthy of being extended to the whole country since it can effectively improve the low-carbon innovation of firms in the pilot areas, thus helping to reduce carbon emissions. Meanwhile, the conclusions of the mechanism analysis suggest that local governments should formulate corresponding green credit policies to alleviate firms’ financing constraints when implementing low-carbon city policies. By doing so, firms can have the ability to carry out R&D investments related to low-carbon development, thus achieving energy conservation and emission reduction.

The limitation of this study is that the number of low-carbon patents may not be the best indicator of the level of low-carbon innovation of a firm because patents vary dramatically in technical difficulties and economic benefits. A new indicator that can better reflect the quality of firms’ low-carbon innovations should be explored in future studies assessing the impact of the low-carbon city pilot policy on firms.

## Supporting information

S1 Data (XLS)
